# Stress and Mood Associations With Smartphone Use in University Students: A 12-Week Longitudinal Study

**DOI:** 10.1177/21677026221116889

**Published:** 2023-01-10

**Authors:** Abigail H. M. Bradley, Andrea L. Howard

**Affiliations:** Department of Psychology, Carleton University

**Keywords:** screen time, smartphones, stress, mood, college students, COVID-19

## Abstract

The current study used device-logged screen-time records to measure week-to-week within-person associations between stress and smartphone use in undergraduate students (*N* = 187; mean age = 20.1 years). The study was conducted during fall 2020 and focused on differences across types of app used and whether accumulated screen use each week predicted end-of-week mood states. Participants uploaded weekly screenshots from their iPhone “Screen Time” settings display and completed surveys measuring stress, mood, and COVID-19 experiences. Results of multilevel models showed no week-to-week change in smartphone hours of use or device pickups. Higher stress levels were not concurrently associated with heavier smartphone use, either overall or by type of app. Heavier smartphone use in a given week did not predict end-of-week mood states, but students who tended to spend more time on their phones in general reported slightly worse moods—a between-persons effect potentially reflecting deficits in well-being that are present in students’ off-line lives as well. Our findings contribute to a growing scholarly consensus that time spent on smartphones tells us little about young people’s well-being.

The study of screen use in relation to mental health and well-being is growing rapidly, with prominent voices raising alarm about screens as likely culprits behind rising mental illness and suicide (Twenge et al., 2018). The surge in smartphone use and contemporaneous increases in mental health issues among adolescents and young adults has paved the way for researchers to examine and understand the association between the two (Sewall et al., 2020). Amidst frequent media attention, research probing effects of screen time on mental health and well-being has been mixed and inconclusive (David et al., 2018; Jensen et al., 2019; Meier & Reinecke, 2020; Orben & Przybylski, 2019; Vuorre et al., 2021). Cross-sectional panel research suggests screen use as a possible risk factor (Twenge et al., 2018), but other panel research shows that the strength of the association between social media and outcomes such as depression, conduct problems, and suicide has remained stable since the mid-2000s (Vuorre et al., 2021).

There have been three primary criticisms of the extant body of work on screen time and well-being. First, that research has been mostly cross-sectional, precluding tests of prospective links between digital technology use and well-being (Coyne et al., 2020). Second, that few studies have attempted to triangulate effects to specific types of use, despite evidence that some apps may be more detrimental to mental health and well-being than others (Andone et al., 2016). Third, that much of the work to date is based on self-reported screen time, which has been shown to be unreliable (Sewall et al., 2020) and weakly correlated with device-logged measures of use (Parry et al., 2021). Here, we addressed these criticisms in a 12-week longitudinal design tracking undergraduates’ stress, mood, and smartphone screen use from September to December of the Fall 2020 academic semester. We used device-logged screen time records to measure week-to-week associations between stress and smartphone use, focusing on differences across types of app used. We also prospectively tested whether accumulated screen use each week, across types of app, predicted end-of-week mood states.

## Undergraduate Stress and the COVID-19 Context

University students are often a focus of research concerning mental health and well-being as they typically face a variety of stressors, including academic expectations, pressure to succeed, postgraduation plans, juggling finances, relationships with family and friends, and overall health (Beiter et al., 2015). Managing these expectations likely contributes to elevated symptoms of ill mental health commonly observed in students, with nearly one third of first-year undergraduates meeting screening criteria for depressive, anxiety, and substance use disorders (Auerbach et al., 2018). Distress levels in undergraduates are also twice that of nonstudent peers (Durand-Bush et al., 2015), suggesting that university students are a vulnerable population for poorer mental health and well-being.

Challenges of the university context intensified beginning in March 2020, when countries around the world shut down and governments imposed shelter-in-place orders in an effort to contain the spread of the SARS-CoV-2 virus that became the COVID-19 global pandemic (World Health Organization, 2021). University students were required to adapt mid-semester to drastic measures such as reductions in social gatherings, forced relocations from student residences, disruptions or closures of campus resources (e.g., library, athletic complex, study halls, and mental health services), and a total shift to online remote learning (Kecojevic et al., 2020). Retrospective reports indicate that students experienced higher stress levels and feelings of isolation during asynchronous online learning compared with a typical face-to-face learning environment (Besser et al., 2022). Several studies found that student stress levels had increased because of COVID-19 (Howard et al., 2022; Patterson et al., 2021; Prowse et al., 2021; Son et al., 2020), and 42.2% of U.S. undergraduates agreed that the pandemic significantly increased their stress levels (American College Health Association, 2020a). In a sample of undergraduates surveyed between May and August of 2020, 61.5% reported that COVID-19 had a moderate to extreme impact on their stress levels (Prowse et al., 2021). Among students attempting to access mental health services, 60% said that the circumstances of the pandemic made it more difficult to access care (American College Health Association, 2020b).

Considerable attention has been focused on screen time as a source of elevated distress during the pandemic (Browning et al., 2021; Richtel, 2021; Smith et al., 2020), punctuated by numerous anecdotal reports of people experiencing shock at the increase in their own smartphone and social media use. In a community sample of emerging adults, self-reported recreational screen time increased by 2.6 hr per week, on average, during the pandemic compared with 2 years earlier (Wagner et al., 2021). In an effort to cope with COVID-19 stress, 79.2% of undergraduate students reported turning to social media—which was correlated with reports of more negative effects of COVID-19 on stress—and 60.4% reported connecting with friends and family using video chat—which was uncorrelated with negative effects of COVID-19 on stress (Prowse et al., 2021).

## Smartphone Use in Longitudinal Research

Given the restrictions on daily life imposed by the pandemic, heightened screen time is inevitable and, in and of itself, unremarkable. If more time spent using smartphones—or specific types of apps—is harmful, it should reliably predict reductions in well-being over time. Longitudinal studies to date show that heavier self-reported social media use does not predict later depressive symptoms year to year (Heffer et al., 2019), nor do device-logged screen time and social media time predict depression, anxiety, or suicidal ideation month to month (Sewall et al., 2021). An 8-year longitudinal design that tracked participants from early adolescence to emerging adulthood found no associations between time spent using social media and mental health, even when participants used more social media than their own cross-time averages (Coyne et al., 2020).

The lack of associations between smartphone use and well-being in longer-term longitudinal studies raises the possibility that day-to-day smartphone screen use effects are time limited and may not accumulate, necessitating designs that collect reports on shorter time scales. For example, one study that surveyed adolescents six times per day for a week detected several time-specific (within-person) associations between screen time and feeling happy, but no average (between-persons) associations (Beyens et al., 2020). On short time scales such as days or weeks, stronger reactions to stressful events at school, work, or home are prognostic of long-term problems of well-being, including higher mortality risk in people with a chronic illness (Chiang et al., 2018) and higher risk of a later depression or anxiety disorder (Charles et al., 2013). The fall semester of the first full academic year occurring during a global pandemic presented a unique opportunity to measure at a more fine-grained level how undergraduate students might be using screens to manage stress. In the present study, we assessed concurrent and prospective week-to-week associations between students’ smartphone screen use and their stress and mood states.

## Smartphone Use Across Specific Types of App

Smartphones are a central feature of daily life. In Canada, 97.9% of young people between the ages of 15 and 24 own a smartphone (Statistics Canada, 2018), as do 96% of 18- to 29-year-olds in the United States (Pew Research Center, 2021). In addition to basic phone, Web browsing, and text-messaging services, people use smartphones for social media, games, news, productivity, and GPS navigation, among other tools (Iyengar et al., 2020). Given the ubiquity of smartphones and the breadth of applications in use, overall *screen time*—the amount of time spent interacting with screens over a specified time frame (Orben, 2020)—is not a useful stand-alone measure. The most comprehensive evidence to date indicates that screen time is associated, on average, with lower well-being in young people but that the effect size is too small to be practically meaningful (Hoare et al., 2016; Orben, 2020; Tang et al., 2021). Indeed, Orben and Przybylski (2019) showed that in large, nationally representative data, *wearing glasses* was more strongly associated with reduced well-being than screen time. Consequently, a priority for new research is to describe the nature and quality of young people’s smartphone use and to identify which narrower, specific uses of screens (social media, entertainment, communication, productivity) are linked to well-being, if any.

The few studies that have tested associations between use of specific apps and well-being confirm heterogeneity in smartphone screen time. In adolescents followed from age 13 to 20, periods of higher self-reported time spent on social media co-occurred with periods of elevated depression and anxiety symptoms (Coyne et al., 2020), though these associations may have been more strongly influenced by passive browsing or scrolling compared with active engagement with social media (e.g., Frison & Eggermont, 2017; Verduyn et al., 2015). For undergraduates, social networking and photo-sharing apps accounted for a third of all device usage, and more self-reported time spent on Instagram was associated with more self-reported depression and anxiety (David et al., 2018). However, in that study, the same size effect was found for more time spent on the Maps application, whereas time spent on book-reading apps was associated with less depression and anxiety. Likewise, more text messaging among teens at high risk of mental illness was associated with fewer depressive and anxious symptoms in a daily diary study (George et al., 2018). These mixed findings support the notion that some kinds of screen use may be more detrimental to mental health and well-being than others. In the present study, we measured smartphone screen time separately across types of frequently used apps (e.g., social networking, text messaging, entertainment and games, productivity) to test week-to-week associations between students’ smartphone use and their stress and mood states.

## Device-Logged Reports of Smartphone Use

A dominant theme in the literature on young people’s screen use is that screen time and social media use worsen mental health and well-being, including increasing stress and anxiety (e.g., Beranuy et al., 2009; Lepp et al., 2014; Thomée et al., 2011). Of the studies making such claims, the vast majority rely on self-report measures to quantify types of screen use—now shown to be highly variable, biased, and unreliable (David et al., 2018; Parry et al., 2021; Vuorre et al., 2021). Previous work comparing instances of self-reported screen time with device-logged reports (e.g., Apple’s Screen Time application) found that participants often underestimate their overall usage (Andrews et al., 2015) and surprisingly overestimate their time spent on social media sites (e.g., Facebook, Instagram, TikTok; Sewall et al., 2020). In a recent meta-analysis, fewer than 10% of screen time self-reports were within 5% of the associated device-logged time, suggesting that people’s personal estimates of screen time usage are rarely accurate (Parry et al., 2021). Studies that have found evidence linking lower levels of well-being to heavier screen time have typically employed self-report measures (e.g., Griffioen et al., 2020; Pantic et al., 2012; Twenge et al., 2018; Woods & Scott, 2016). In studies that correlate device-logged screen time with well-being measures, however, low to nonexistent correlations are more typical (Ellis et al., 2019; Sewall et al., 2020, 2021; Shaw et al., 2020). In the present study, we measured device-logged reports of screen time, device pickups, and usage across types of app by asking participants to upload screenshots from their iPhone Screen Time settings each week across the fall semester.

## The Current Study

The current study addressed the tripartite criticism of existing research on screen time and well-being in a 12-week longitudinal study of undergraduates who provided device-logged reports of their smartphone use. We tested concurrent and prospective associations with stress and mood states, focusing on differences across types of app. We had four aims. First, we tested whether there were systematic weekly changes from September to December in four indicators of smartphone screen use: (a) overall daily hours spent on smartphone, (b) daily hours spent on frequently used apps, (c) overall daily number of pickups, and (d) daily pickups associated with frequently used apps. Second, we tested whether general stress (e.g., at home, work, and school over the past week) and stressful experiences related to COVID-19 (event cancellations) were associated with each of the four indicators of screen use week to week. Third, we tested whether associations between stress and app-specific smartphone screen use differed across types of app most frequently used (e.g., social networking, text messaging, entertainment, productivity). And finally, we tested whether cumulative screen use across the week for each smartphone screen use indicator predicted daily mood states reported at the end of each week (feeling happy, relaxed, irritated, and stressed), also allowing for differences across types of app. Analyses were preregistered (https://osf.io/a9xpg). Data, analysis code, and other materials are available on our OSF project page: https://osf.io/2gy63/.

Data for this study were gathered as part of a multipurpose pilot project following undergraduate students in the fall semester of 2020, nearly 6 months after the beginning of the COVID-19 pandemic. Aims of that project were to test time-varying associations between stress and cannabis use, to pilot-test a series of measures related to alcohol use, and to pilot-test the file-upload question format in Qualtrics to gather screenshots of smartphone time use. We had no immediate plans involving the screen time data and developed the current study plans after observing an optimistic adherence rate across extensive repeated measures.

## Method

### Participants and procedure

Participants were 187 undergraduate students under 30 years of age who were attending a Canadian university in the fall of 2020 and who used a smartphone equipped with Apple’s iOS operating system. Participants were recruited from two sources: (a) the Sona Systems undergraduate psychology participant pool at our university and (b) Prolific.co online research participation services. We used built-in prescreening data to identify Prolific participants who were residing in Canada and currently undergraduate students. Panelists who met these criteria received access to a custom eligibility screening survey. Only those who declared that they owned a smartphone, were under 30, and were current undergraduate students were added to our prospective participant list. Participants recruited from both sources next completed a 20-min intake survey after reading a brief study description and providing informed consent. Participants were next invited to complete weekly surveys for up to 12 weeks spanning the fall semester. We selected this time frame because iPhone Screen Time summaries are available in weekly intervals and to minimize respondent burden without sacrificing detailed time-varying data.

Sona participants (*n* = 133) received course credit for completing the intake survey and additional credits for every 2 weekly surveys they completed, with bonus credits awarded for completing at least 10 weekly surveys. Prolific participants (*n* = 54) received $5 for completing the intake survey and $1 for each weekly survey completed, with an additional $1 bonus payment each time a participant completed 3 weekly surveys. Participants were enrolled on an ongoing basis through mid-October. Retention varied, with 47 participants completing at least eight surveys, 73 completing between four and seven surveys, and 32 completing two or three surveys (35 people participated just once). In total, we recorded 973 weekly responses. A detailed breakdown of weekly participation rates is available on our OSF project page.

Participants were 17.64 to 29.67 years old (*M* = 20.11, *SD* = 2.51) and mostly women (80.9%; *n* = 152); 18% identified as male (*n* = 34), and one participant identified as both female and male. Participants were from all years of study: 50.5% (*n* = 95) were first-year students, 26.7% (*n* = 50) were second-year students, 12.3% (*n* = 23) were third-year students, and 11.8% (*n* = 22) were fourth/final-year students. Most participants (67.9%; *n* = 127) reported that one or more parents had a university degree. Participants identified with a range of racial/ethnic backgrounds: 61% (*n* = 114) self-identified as White, 12.3% (*n* = 23) as Southeast Asian, 6.4% (*n* = 12) as South Asian, 5.9% (*n* = 11) as Black, 5.9% (*n* = 11) as Indigenous, 3.2% (*n* = 6) as West Asian/Middle Eastern, 1.6% (*n* = 3) as Latin American, and 3.7% (*n* = 7) as mixed race/ethnicity. Living situations included campus residence (15.0%; *n* = 28), living with parents (61.0%; *n* = 114), and living off campus, either alone or with roommates (24.1%; *n* = 45).

### Measures

#### Device-logged reports of screen time

**Fig. 1.**
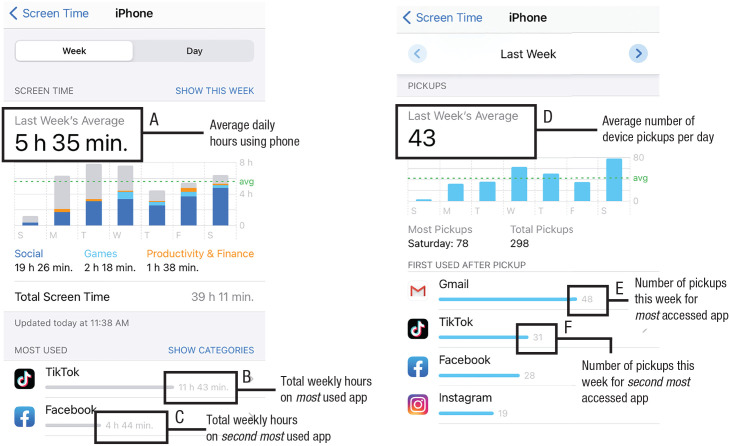
Sample screenshots from the iPhone Screen Time app showing overall daily phone hours (a), daily hours spent on the most frequently used app (b) and second-most frequently used app (c), overall daily phone pickups (i.e., instances of the user interacting with an app on their device after it was sitting idle; d), and the number of times per day the smartphone was picked up to access the most frequently used app (e) and second-most frequently used app (f). Weekly totals were converted to daily averages.

At each weekly survey, available from Sunday through Tuesday morning, participants were asked to upload two screenshots from their Screen Time application (a preinstalled app located in an iPhone’s settings; see Fig. 1). The Screen Time application provides a report showing how the device was used, including names of apps that were opened, hours of use (global and app specific), and numbers of times the device was picked up (Apple Support, 2021). We provided participants with a detailed how-to video instructing them where to go, how to take a screenshot of their usage from the previous week, and how to upload their screenshots as survey responses (video available on our OSF project page).

Volunteer research assistants extracted six measures of screen use from each person’s screenshots: overall daily phone hours (Fig. 1a), daily hours spent on the most frequently used app (Fig. 1b) and second-most frequently used app (Fig. 1c; both converted from weekly hours), overall daily phone pickups (i.e., instances of the user interacting with an app on their device after it was sitting idle; Fig. 1d), and the number of times per day the smartphone was picked up to access a specific app, both the most frequently accessed app (Fig. 1e) and the second-most frequently accessed app (Fig. 1f; both converted from weekly pickups). Frequent apps varied within person depending on the metric. A person’s most frequently used app (in minutes/hours of use per week) often differed from their most frequently accessed app (in number of pickups). Figure 1 shows that the sample participant most often picked up their phone to use Gmail, but they spent the most time on TikTok compared with any other single app.

For frequent-use measures, we classified each app as related to *entertainment and gaming* (e.g., YouTube); *social networking* (e.g., Instagram, TikTok, text messaging); *information, reading, and productivity* (e.g., Safari, Duolingo); and *other*. For frequent-access measures, we separated social networking into two categories—*social media* (e.g., Instagram) and *text messaging*—given the high volume of reports showing a text-messaging app to be the first app accessed on a pickup. We include final classifications for each app coded in our data on our OSF project page.

#### Stressful experiences

The Daily Inventory of Stressful Events (DISE; Almeida et al., 2002) assessed overall stress experienced at home, school, work, and other settings. Participants were asked whether certain types of events that occurred in the past week were stressful (e.g., “Did anything happen at school last week that most people would consider stressful?”). If participants responded “yes,” stress severity was measured using a 4-point Likert scale (e.g., “How stressful was this for you?”; 1 = *not at all*, 2 = *not very*, 3 = *somewhat*, 4 = *very*). Participants who responded “no” or for whom the setting was not applicable (e.g., did not work in the past week) were assigned a 0 on the corresponding severity item. A mean stress score was given to each participant by averaging responses to each of the four questions. Stress scores could range from 0 (experienced no stressful events) to 4 (experienced stress in all four settings at very high levels; Wong et al., 2012). During scale development, the interrater reliability of the DISE ranged from .66 to .96, which is considered good to excellent (Wethington & Almeida, 2009).

Participants were also asked each week whether any events had been postponed or cancelled within the past week (0 = *No*, *n* = 770; 1 = *Yes*, *n* = 187) and whether they received a COVID-19 test within the past week (0 = *No*, *n* = 943; 1 = *Yes*, *n* = 24). Event cancellations or postponements were reported on 19.5% of weeks. At least one postponement/cancellation was reported by 103 (55%) participants (range = 1–5). COVID-19 tests were reported on just 2.5% of weeks by 19 (10%) participants (range = 1–3). Given the very low number of reported tests for COVID-19, we did not have sufficient variability to conduct our preregistered analyses of past-week COVID-19 tests as a time-varying covariate of screen use. We proceeded with event cancellations and stress scores.

#### Mood

Positive and negative affect were examined each week by asking participants to rate how they felt on four common affect states: “happy,” “relaxed,” “irritated,” and “stressed.” The question was displayed as, “On Saturday, to what extent did you feel…” Each mood was rated on a 5-point scale from 1 (*very slightly/not at all*) to 5 (*extremely*). This was an adaptation of the Positive and Negative Affect Schedule–Short Form (Thompson, 2007) implemented in other ecological momentary assessment (EMA) research as a means to reduce response burden (O’Donnell et al., 2019).

### Missing data

Week-to-week participant retention and incomplete screenshot uploads were two sources of missing data in this sample. Out of 973 weekly responses recorded, 148 (15.2%) and 171 (17.6%) screenshots pertaining to past-week phone hours and past-week pickups, respectively, were missing. Of past-week phone hours, 133 screenshots were unusable and 15 had not been submitted by participants. Of past-week pickups, 151 screenshots were unusable and 20 were not submitted. A screenshot was deemed unusable if it did not include a past-week summary (e.g., the screenshot contained only 1–2 days’ worth of screen use) or was a duplicate (e.g., a screenshot from one week was uploaded more than once). Out of 973 weekly responses, 860 included data from at least one screenshot.

To evaluate the plausibility of a missing at random (MAR) mechanism, we counted for each person the numbers of weeks in which they provided data on overall hours of smartphone use and overall daily pickups. We used each of these counts as person-level predictors of key repeated measures variables (weekly stress, event cancellations, and mood), and we tested for mean differences in counts of valid smartphone use data across demographic measures (gender, year of study, racial/ethnic identity, living arrangements, data source). Note that we did not include a strategy for working with missing data within our preregistration, and this strategy was ad hoc.

Participants who provided more weeks of data did not differ from participants who provided fewer weeks of data on any measures of smartphone use or on any measures of mood. Having more weeks of data was associated with slightly lower stress scores—about one twentieth of a standard deviation less for each additional week of data—and with a slightly lower odds of reporting any event cancellations because of COVID. One third of observations reported by participants with 4 or fewer weeks of data included reports of event cancellations in the past week compared with just 14% of observations reported by participants with 8 or more weeks of data. We found no differences in the numbers of weeks of data provided by participants of different genders, ethnicities, or living situations. Participants recruited from Prolific.co provided on average 1.6 more weeks of data compared with participants recruited from our local Sona pool, and fourth-year students provided on average 2.4 more weeks of data compared with first-year students (there were no other differences in weeks of provided data by year of study).

A third source of missing data was a survey error that resulted in 42 participants across 139 occasions viewing a random selection of three of four stress-scale items instead of the full four-item scale. Because the items participants saw were randomly selected, this error induced a missing completely at random (MCAR) mechanism, and we computed mean stress scores by averaging available items. Overall, we judged the impact of missing data in the present study to be minimal and proceeded with analyses using full-information maximum-likelihood estimation to retain available cases.

### Analysis plan

Analyses for this study were performed using R software (R Core Team, 2021) and the packages *lme4* (Bates et al., 2015), *lmerTest* (Kuznetsova et al., 2017), *dplyr* (Wickham et al., 2021), *jtools* (Long, 2020), *car* (Fox & Weisberg, 2019), and *emmeans* (Lenth, 2021).

We used multilevel linear models with random intercepts and (where possible) random slopes to estimate associations between smartphone use (hours, pickups) and stress and mood. Tests proceeded in three stages. First, we estimated weekly change over time in all six measures of smartphone use, considering linear, quadratic, and spline functions to approximate the best functional forms of change over time. Second, we estimated time-varying (within-person) associations between past-week stress (perceived severity and event cancellations) and each of the six measures of smartphone use. Interactions between time-varying stress and app category were tested in a separate step. Time-varying stress scores were person-mean centered (Curran & Bauer, 2011; Howard, 2015), and all models included participants’ average levels of stress across all weeks. We did not explicitly note this in our preregistered plan, but it is customary to include person means, and in the case of dummy-coded event cancellations, the person mean is needed to obtain an estimate of a time-varying effect that is fully disaggregated from its average or person-level counterpart (Yaremych et al., 2021). A key advantage of estimating time-varying and average effects separately is the ability to rule in or out potential sources of variance. For example, a time-varying effect of stress on hours of use tests whether weeks of higher than usual stress for a given person are linked to more hours of smartphone use, irrespective of that person’s typical levels of stress and smartphone hours. The average effect is needed to determine whether a person who typically reports higher stress tends to spend more time on their phone.

Third, we estimated prospective time-varying associations between past-week smartphone use and each of the four end-of-week mood states. Interactions between time-varying smartphone use and app category were tested in a separate step. Time-varying smartphone hours were person-mean centered, and participants’ average levels of smartphone use across all weeks were included in models. Our preregistered plan called for tests of interactions between time-varying covariates and gender, but given the small number of men in the sample, we elected not to proceed with these analyses.

Finally, our analyses involved a large number of significance tests and we adjusted for multiple comparisons using the Benjamini-Hochberg false-discovery rate procedure (Benjamini & Hochberg, 1995) against a nominal α of .05. To balance risks associated with inflating Type II errors, we performed corrections within several smaller “families” of related tests rather than correcting for study-wide alpha inflation. We defined one family of tests comprising all six models for change over time in smartphone use, correcting *p* values from the final models only. The smallest *p* value required for statistical significance in this family was .0083. To test time-varying associations between stress and smartphone use, we defined four families, each comprising 14 tests: weekly stress associations with daily phone hours, weekly stress associations with daily phone pickups, weekly event cancellation associations with daily phone hours, and weekly event cancellation associations with daily phone pickups. To test prospective effects of smartphone use on end-of-week mood states, we defined eight families, each comprising 14 tests: daily phone hours predicting each of the four mood states and daily pickups predicting each of the four mood states. The smallest *p* value required for statistical significance within each of these 14-test families was .0036.

Sample size in this study was limited by a combination of financial constraints and time. We conducted a power analysis and determined that a sample of 187 participants producing around 900 weekly responses was adequately powered to detect a standardized effect size of just 0.16 from a continuous predictor variable with a small random slope at a nominal Type I error rate of .05. We conducted several follow-up simulations to evaluate power to detect the same-sized effect within a family of 14 tests as described above. Power to detect at least one effect out of 14 at *p* < .0036 is ample if all effect sizes within the family are the same (0.16) or are a mix of sizes ranging from 0 to 0.16 (estimated power > .99 and .95, respectively). Power was modest (.50) in the conservative case in which just one effect in the family is 0.16 and all others are exactly zero in the population. Reproducible simulation code with five power scenarios is available on our OSF project page.

## Results

**Fig. 2.**
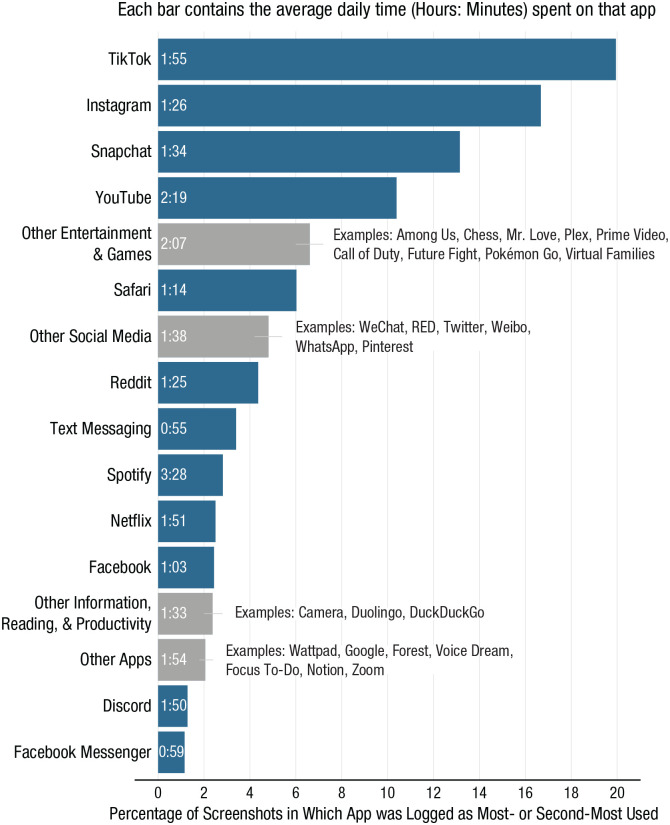
Most and second-most frequently used iPhone apps based on device-logged reports of past-week screen time taken September 8 to December 14, 2020.

Table 1 shows means, standard deviations, and correlations for measures of smartphone use, stress, and mood. Figure 2 shows a ranked listing of frequently used apps. In total, 88 different apps were logged on participants’ smartphones as either most or second-most frequently used, but in 60% of records, participants used TikTok (20%), Instagram (17%), Snapchat (13%), and YouTube (10%) most or second-most often. On average, students used their smartphones for 6 hr 53 min per day and picked up their phones 113 times. Their most and second-most frequently used apps accounted for 2 hr 14 min and 1 hr 12 min of use per day on average, respectively. Students picked up their phones 28 times per day for their most accessed app, and 11 times per day for their second-most accessed app. A supplemental figure showing distributions of usage hours by app is available on our OSF project page. Device pickups are not pictured, but students typically picked up their phones to access a social media app or text messaging. Snapchat accounted for 26% of records of apps that students accessed most or second-most frequently immediately after unlocking their phones; Messages (iPhone’s built-in text-messaging app) accounted for 24%, and Instagram accounted for 15%.

**Table 1.** Summary Statistics and Correlations for Study Variables

Variable	*M* (*SD*)	ICC	Pearson correlations										
			1	2	3	4	5	6	7	8	9	10	11
1. Daily phone hours	6.89 (2.69)	.83	−										
2. Daily hours, most frequent app	2.23 (1.37)	.68	**.77**	−									
3. Daily hours, second-most frequent app	1.2 (0.66)	.63	**.76**	**.59**	−								
4. Daily number of pickups	113.34 (52.38)	.86	**.04**	−.08	−.03	−							
5. Daily pickups, most accessed app	27.86 (24.19)	.87	**.10**	**.09**	.11	**.80**	−						
6. Daily pickups, second-most accessed app	11.27 (6.63)	.72	**.03**	−.05	.04	**.56**	**.33**	−					
7. Weekly mean stress	1.13 (0.98)	.45	**.17**	**.11**	.04	**.06**	.02	.11	−				
8. Event cancellations	.20 (.40)	.28	.06	.08	.02	.01	.01	−.03	**.17**	−			
9. End-of-week happy mood	3.26 (1.11)	.37	**−.17**	−.20	**−.17**	.23	.08	.17	**−.19**	−.02	−		
10. End-of-week relaxed mood	2.86 (1.12)	.23	−.15	−.19	−.12	.14	.02	.12	**−.23**	−.02	**.65**	−	
11. End-of-week irritated mood	2.5 (1.29)	.36	**.26**	.21	.14	.06	.11	.05	**.34**	.07	**−.41**	**−.44**	−
12. End-of-week stressed mood	3.0 (1.32)	.30	.17	.12	.09	−.01	.02	−.05	**.40**	.04	**−.50**	**−.60**	**.60**

Note: Correlations in boldface are statistically significant (*p* < .05). ICC = intraclass correlation coefficient.

To contextualize effect sizes in the analyses that follow, we supplemented the person-level summary statistics in Table 1 by calculating within-person variability in daily smartphone hours and numbers of pickups. A typical student’s daily hours of smartphone use varied by 58 min across reports (intraindividual *SD* = 0.97 hr) with a range of 2 hr per day between their heaviest and lightest using weeks. A typical student’s daily phone pickups varied by 17 (intraindividual *SD* = 16.79), with a range of 32 daily pickups between their heaviest and lightest using weeks.

### Week-to-week change in smartphone screen use

**Fig. 3.**
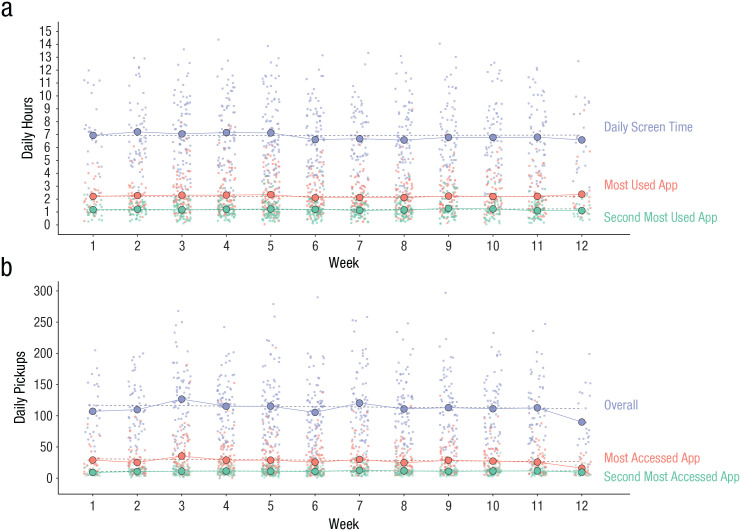
Trends over time in average daily smartphone hours and pickups from the week of September 8 through December 14, 2020. Panel (a) shows average daily hours, daily hours spent on the most frequently used app, and daily hours spent on the second most frequently used app. Panel (b) shows average daily pickups, daily pickups corresponding to the most frequently accessed app, and daily pickups corresponding to the second most frequently accessed app. Model-implied trends are shown as dashed lines. For each week, smaller circles indicate individual data, and larger circles indicate means.

Figure 3a shows average daily hours students spent on their iPhones from the week of September 8 through December 14, 2020. Trends for overall daily hours, daily hours spent on their most frequently used app, and daily hours spent on their second-most frequently used app were largely stable over time. The linear slopes for study week were trivially small and not significantly different from zero (range: *b* = −0.003 to 0.01), implying mean change in screen time of no more than 36 s per week, on average. Likewise, trends for overall daily pickups, number of pickups corresponding to the most frequently accessed app after pickup, and number of pickups corresponding to the second-most frequently accessed app after pickup showed stability (see Fig. 3b). Linear slopes for study week were also no different from zero (range: *B* = −0.51 to 0.04), implying mean change of up to half a pickup each week. Likelihood-ratio tests comparing models containing only a linear trend over time to quadratic and spline functions showed no improvement in fit when more complex functions of time were permitted. Given the lack of evidence for systematic week-to-week change, time trends were excluded from subsequent models.

### Associations between weekly stressful experiences and screen use

Table 2 shows results of 10 models testing the time-varying associations between self-reported stress and screen use. Students’ weekly stress scores were centered around their own mean stress score across all weeks. Models also included students’ average stress scores to test between-persons effects of average stress levels on average screen use. For the six models shown in the first two rows (“Weekly Stress—Model 1: overall”), effects were nonsignificant and trivially small in size. For example, during weeks when a student’s stress levels were 1 standard deviation higher than usual, they would be expected to spend an extra 7 min per day on their smartphone (0.98 × 0.117 × 60 = 6.88 min) and pick up their phone an extra 2 times per day (0.98 × 1.867 = 1.8 pickups). No estimates were significantly different from zero.

**Table 2.** Time-Varying Effects of Weekly Stress and Weekly Event Cancellations on Daily Hours Students Spent on Smartphones and Times per Day Students Picked Up Their Smartphones (Overall and Separately by Type of App)

Model and variable	Daily phone hours			Daily phone pickups		
	Total	Most frequently used app	Second-most frequently used app	Total	Most accessed app	Second-most accessed app
	Estimate *b*(*SE*)	Estimate *b*(*SE*)	Estimate *b*(*SE*)	Estimate *b*(*SE*)	Estimate *b*(*SE*)	Estimate *b*(*SE*)
Weekly stress						
Model 1: overall						
Time-varying stress	0.125 (0.059)*	0.067 (0.052)	−0.019 (0.022)	1.867 (1.061)	0.635 (0.481)	0.333 (0.193)
Average stress	0.379 (0.218)	0.098 (0.106)	−0.010 (0.051)	−1.508 (4.274)	−1.508 (2.032)	0.528 (0.531)
Model 2: time-varying by app						
Entertainment and games		0.253 (0.103)*	−.054 (0.050)		−0.825 (3.309)	0.784 (0.763)
Social networking		0.020 (0.058)	−.004 (0.027)			
Text messaging					0.702 (0.551)	0.383 (0.287)
Social media					0.853 (1.343)	0.373 (0.353)
Info/read/productivity		−0.211 (0.227)	−.031 (0.080)		−2.890 (2.993)	−0.159 (0.534)
Other		0.354 (0.314)	−0.007 (0.170)			
Weekly event cancellations						
Model 1: overall						
Time-varying cancellations	0.132 (0.166)	0.148 (0.116)	0.090 (0.066)	−0.210 (2.42)	−0.422 (1.125)	−0.425 (0.441)
Average cancellations	−0.212 (0.651)	−0.174 (0.340)	−0.260 (0.163)	8.125 (12.77)	7.578 (5.832)	−2.038 (1.627)
Model 2: time-varying by app						
Entertainment and games		0.251 (0.178)	−0.050 (0.230)		−0.810 (4.76)	1.484 (1.369)
Social networking		0.100 (0.145)	0.176 (0.138)			
Text messaging					0.547 (1.24)	−0.149 (0.618)
Social media					−6.308 (2.67)*	−0.800 (0.742)
Info/read/productivity		−0.425 (0.401)	0.364 (0.351)		7.636 (5.25)	−1.334 (1.21)
Other		−1.762 (0.925)	−1.312 (0.887)			

* *p* < .05, not statistically significant after correction for multiple testing.

Table 2 also shows results of six models testing the time-varying associations between event cancellations and screen use. Models included dummy-coded event cancellation scores each week and adjusted for students’ proportion of event cancellations across all weeks (0 = student did not report any event cancellations, 1 = student had at least one event cancelled on every week they provided data). For the six models shown under “Weekly Event Cancelations—Model 1: Overall,” effects were again nonsignificant and small in size. For example, during weeks that a student reported a cancelled event because of COVID-19, they would be expected to spend an extra 8 min per day on their most frequently used app (0.132 × 60 = 7.92 min) and pick up their phone one less time every 5 days (1/−0.210 = 4.76 days).

**Fig. 4.**
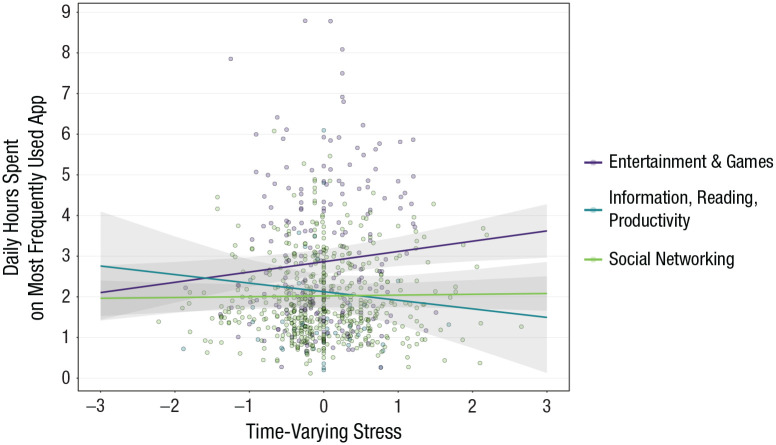
Time-varying associations between self-reported stress and daily hours students spent on their most frequently used app (categorized as social networking, entertainment and games, or information, reading, and productivity). Lines represent model-implied simple slopes for each category of app, pictured with 95% confidence bands.

We considered that associations between stressful experiences and smartphone screen use might be different across categories of app. Adding Time-Varying Stress × App Category interaction terms to a model containing only main effects did not reduce the model log likelihood—for most frequently used app categories, χ^2^(3) = 5.87, *p* = .12; for second-most frequently used app categories, χ^2^(3) = 0.83, *p* = .84. Table 2 (“Weekly Stress—Model 2: Time-Varying by App”) shows model-estimated effects for each category of app. There were no associations between weekly stress and smartphone use that were app specific. The only effect we detected, which did not survive correction for multiple testing, was the time-varying association between self-reported stress and daily hours students spent on their most frequently used app for the category of entertainment and games. Figure 4 illustrates the modest size of this effect: During weeks that students most often used entertainment and gaming apps (e.g., YouTube, Netflix, Among Us), students whose stress levels were 1 standard deviation higher than usual spent an extra 15 min per day on entertainment and gaming apps (0.98 × 0.253 × 60 = 14.9 min). Over the course of a week, this translated to an extra three to four half-hour episodes of a YouTube show or 1.75 more hours playing Among Us. Associations for other categories of most frequently used apps were not statistically significant, providing no evidence that higher stress co-occurs with more time spent on social networking or other types of apps.

For weekly event cancellations, adding Time-Varying Event × App Category interaction terms also did not reduce the model log likelihood over that of a model containing only main effects—for most frequently used app categories, χ^2^(3) = 6.73, *p* = .08; for second most frequently used app categories, χ^2^(3) = 4.27, *p* = .23. As shown in Table 2 (“Weekly Event Cancellations—Model 2: Time-Varying by App”), none of the app-specific associations between event cancellations and smartphone use were statistically significant.

We also tested the contributions of interactions with app category for time-varying stress and event cancellations linked to daily pickups and again found no reductions in model loglikelihoods (χ^2^ values ranged from 1.24 to 6.76). After correction for multiple testing, none of the associations we tested between self-reported stress or event cancellations and smartphone pickups were statistically significant for any category of app most or second-most frequently accessed immediately after pickup. The sole effect we observed (that did not survive correction) suggested that during weeks when students reported COVID-related event cancellations, they picked up their phones to access social media apps about 6 fewer times per day.

### Prospective associations between screen use and end-of-week mood states

Table 3 shows results of 40 models testing prospective effects of screen use and pickups over the past week (Sunday to Saturday) on students’ mood states at the end of the week (Saturday). Students who used their phones more than usual in a given week reported feeling less happy at the end of the week (see Table 3, “Total Daily Phone Hours”). However, this association was small, not present for any other mood state, and did not survive correction for multiple testing. Specifically, a student would need to use their phone for 6.8 hr more than usual each day to record a half-standard-deviation reduction in happiness at the end of the week (1.11/2 ÷ −0.082 = 6.8). As noted earlier, a typical student’s daily use fluctuates by less than an hour. For other mood states, people who tended to use their phones more often on average reported feeling less relaxed, more irritated, and more stressed on average across all weeks, though these were also small associations and only the latter two survived correction for multiple testing. The largest effect was for feeling irritated—students who generally used their phones an extra 5.4 hr per day than the average student reported feeling one half standard deviation more irritated than the average student (1.29/2 ÷ 0.120 = 5.4).

**Table 3.** Prospective Effects of Screen Use and Pickups Over the Past Week (Sunday to Saturday) on Students’ Mood States at the End of the Week (Saturday)

Model and variable	End-of-week mood state			
	Happy *b*(*SE*)	Relaxed *b*(*SE*)	Irritated *b*(*SE*)	Stressed *b*(*SE*)
Total daily phone hours				
Time-varying hours	−0.082 (0.031)*	0.007 (0.035)	0.056 (0.036)	−0.013 (0.038)
Average hours	−0.049 (0.026)	−0.059 (0.023)*	0.120 (0.028)*	**0.086 (0.028**)*
	Daily hours: most frequent app			
Model 1: overall				
Time-varying hours	−0.039 (0.063)	−0.005 (0.052)	0.028 (0.053)	0.002 (0.071)
Average hours	−0.121 (0.053)*	**−0.154 (0.048**)*	**0.198 (0.061**)*	0.152 (0.060)*
Model 2: time-varying by app				
Entertainment and games	0.012 (0.097)	0.072 (0.076)	−0.016 (0.078)	−0.121 (0.108)
Social networking	−0.123 (0.091)	−0.127 (0.089)	0.123 (0.092)	0.106 (0.109)
Information/reading/productivity	−0.220 (0.315)	0.103 (0.315)	0.315 (0.330)	0.019 (0.369)
Other	0.473 (0.295)	−0.350 (0.568)	−0.642 (0.308)*	0.094 (0.353)
	Daily hours: second-most frequent app			
Model 1: overall				
Time-varying hours	−0.170 (0.094)	0.039 (0.109)	0.068 (0.110)	−0.070 (0.117)
Average hours	−0.298 (0.118)*	−0.321 (0.105)*	0.300 (0.137)*	0.266 (0.136)
Model 2: time-varying by app				
Entertainment and games	−0.338 (0.193)	−0.0002 (0.177)	0.114 (0.178)	−0.271 (0.238)
Social networking	−0.050 (0.161)	0.169 (0.173)	−0.059 (0.176)	−0.082 (0.201)
Information/reading/productivity	−0.424 (0.280)	−0.198 (0.277)	0.446 (0.281)	0.509 (0.341)
Other	−0.877 (1.218)	0.726 (1.383)	−2.588 (1.39)	−1.410 (1.43)
Total daily phone pickups				
Time-varying pickups	0.0008 (0.0017)	−0.0003 (0.002)	0.0004 (0.002)	−0.0005 (0.002)
Average pickups	0.0052 (0.0012)*	0.0035 (0.002)*	0.0002 (0.0015)	−0.0008 (0.0015)
	Daily pickups: most accessed app			
Model 1: overall				
Time-varying pickups	−0.002 (0.004)	−0.005 (0.004)	−0.0001 (0.004)	−0.003 (0.005)
Average pickups	0.006 (0.003)*	0.0027 (0.0025)	0.003 (0.003)	0.00007 (0.003)
Model 2: time-varying by app				
Entertainment and games	0.006 (0.040)	−0.013 (0.045)	0.014 (0.047)	−0.058 (0.050)
Text messaging	−0.001 (0.004)	−0.005 (0.005)	0.0002 (0.005)	−0.002 (0.005)
Social media	−0.008 (0.012)	−0.009 (0.013)	0.0002 (0.014)	−0.003 (0.015)
Information/reading/productivity	−0.020 (0.022)	−0.019 (0.025)	−0.012 (0.025)	−0.005 (0.027)
	Daily pickups: Second-most accessed app			
Model 1: overall				
Time-varying pickups	0.009 (0.010)	−0.003 (0.011)	−0.002 (0.011)	0.0001 (0.012)
Average pickups	0.029 (0.010)*	0.028 (0.010)*	0.004 (0.012)	−0.015 (0.012)
Model 2: time-varying by app				
Entertainment and games	−0.016 (0.042)	−0.009 (0.045)	0.120* (0.048)	0.0003 (0.050)
Text messaging	−0.003 (0.014)	0.008 (0.015)	−0.026 (0.016)	0.009 (0.018)
Social media	0.036* (0.018)	−0.016 (0.020)	0.007 (0.021)	−0.008 (0.023)
Information/reading/productivity	−0.006 (0.035)	−0.031 (0.039)	0.019 (0.040)	−0.014 (0.046)

Note: Effects in boldface are statistically significant after correction for multiple testing using the Benjamini-Hochberg false-discovery rate method. Effects marked with an asterisk had *p*s < .05 but were not significant after correction.

For hours students spent on their most frequently used app (see Table 3, “Daily Hours: Most Frequent App”), there were no time-varying effects of screen time on end-of-week mood states. Again, only between-persons effects were significant and indicate that students who generally spent more time on their most frequently used apps reported feeling less happy, less relaxed, and more irritated on average across all weeks, again showing small associations (see Supplemental Figure 2 on our OSF project page for a sample visualization). In a supplemental analysis that we did not preregister, we determined that between-persons effects on mood states were not significantly different across categories, with the exception of one effect that survived correction for multiple testing: Averaging over the weeks that students’ screen time logs showed their most frequently used app to be an entertainment or games app, we found that more hours spent on those apps was associated with feeling more irritated (*b* = 0.274, 95% confidence interval [CI] = [0.11, 0.44], *p* = .0013). A 2-hr 21-min higher average daily usage of entertainment or gaming apps was associated with typically feeling one half standard deviation more irritated compared with peers who spent fewer hours on these apps (1.29/2 ÷ 0.274 = 2.35 hr). App-specific associations were not found across weeks when social networking or other apps were used most or second-most frequently, after correction for multiple testing. When averaging over weeks that students’ screen time logs showed their second-most frequently used app to be a social networking app, we noted a consistent direction of effects linking more hours to worse mood states for each of the four moods, though none of the *p* values survived correction for multiple testing. Supplemental Table 2 on our OSF project page shows complete results of these exploratory analyses for between-persons effects of hours spent on the most frequently used app and the second-most frequently used app across four categories of app.

For daily pickups, there were no time-varying effects on end-of-week mood states (see Table 3, “Total Daily Phone Pickups”). Only between-persons effects were significant, and only for feeling happy or relaxed (see Supplemental Figure 3 on our OSF project page for a sample visualization). Recall that a typical student picked up their phone 113 times per day. Averaging over all weeks, students who picked up their phones nearly twice that often—an extra 107 times per day—reported feeling one half standard deviation happier than the average student (1.11/2 ÷ 0.0052 = 106.7).

The effect size was similar for feeling relaxed. For apps most frequently accessed immediately after pickup, there were no time-varying or between-persons associations between more frequent app-specific pickups and end-of-week mood states (Table 3, “Daily Pickups: Most Accessed App”). For apps second-most frequently accessed immediately after pickup, there were no time-varying associations (Table 3, “Daily Pickups: Second-Most Accessed App”), but we observed between-persons effects for feeling happy and relaxed that survived correction for multiple testing (*p*s < .0051). A typical student in a typical week picked up their phone 11 times per day to use their second-most accessed app. Averaging over all weeks, students who picked up their phones an extra 19 to 20 times per day to use a second-most accessed app reported feeling one half standard deviation happier and more relaxed than the average student (1.11/2 ÷ 0.029 = 19.1; 1.12/2 ÷ 0.028 = 20). The identity of each person’s most- and second-most accessed apps change from week to week, but associations between app-specific pickups and end-of-week mood states were not different across categories of app (no app-specific pickup effects were significant after correction for multiple testing).

## Discussion

In the fall of 2020, we asked undergraduates to report their stress and mood each week for up to 12 weeks and to provide screenshots of their iPhones’ past-week Screen Time reports. Our goal was to achieve a finer-grained picture of the relation between smartphone use and well-being by linking stress and mood to smartphone use within specific categories of app in addition to overall hours of use and device pickups. We found that students used their phones nearly 7 hr per day and that this rate of use was consistent week to week across the fall semester. Pre-COVID studies of device-logged smartphone use show that daily use previously ranged from 3 to 5 hr (Andrews et al., 2015; David et al., 2018; Sewall et al., 2020; Shaw et al., 2020). We did not find strong evidence that the hours students spent on their phones each week varied in tandem with stress levels or with COVID-related event cancellations. We also prospectively tested whether screen use during the week predicted end-of-week mood states. A consistent finding from our prospective tests was that only between-persons effects—that is, averages or tendencies to spend more time on one’s smartphone—were associated with mood. Findings from this series of tests suggest that students’ well-being is likely not responsive to week-to-week changes in smartphone use. Rather, students who tend to use their phones a lot tend to feel a little bit less happy, less relaxed, more irritated, or more stressed. Students who tend to pick up their phones a lot feel—if anything—a little bit happier and more relaxed, regardless of which apps they tend to access when they pick up their phones.

### Limited support for app-specific associations between stress or mood and smartphone use

Looking across all tests of app-specific effects in the present study, we found little evidence for any robust links between smartphone use and stress or mood that differed by app. In the category of entertainment and gaming app use, we observed one time-varying association between stress and daily hours spent on the most frequently used app, which did not survive correction for multiple testing, and another (not preregistered) between-persons association linking heavier hours on entertainment and gaming apps to greater average feelings of irritation. These effects are weak but consistent with other research showing that students spend more time watching videos or gaming when they are feeling stressed. In one study, participants reported binge-watching TV shows as a way to distract themselves, to temporarily escape reality, and to manage stress (Vaterlaus et al., 2019). Video and gaming content may alternatively generate some feelings of stress when there is a high investment in the content. Binge-watching shows, for example, can be highly emotional and associated with feelings of guilt or regret (Wang, 2019). Given the distribution of app usage shown in Figure 1, few students in this sample were likely binge-watching shows on their phones, but the potential for entertainment and gaming apps to serve stress-inducing and -relieving purposes is plausible (e.g., Reinecke & Hofmann, 2016). One recent study found that gaming had many positive effects on players’ well-being during the COVID-19 pandemic by acting as a stress reliever and providing a mentally stimulating escape from the effects of lockdowns (Barr & Copeland-Stewart, 2021).

Crucially, we observed no time-varying effects of stress and screen hours during weeks when students frequently used a social networking app. This contradicts media reports and much of the pre-COVID screen time literature that relies on self-reports of screen time and suggests that heavier social networking contributes to worse well-being (Huang, 2017; Karim et al., 2020; Kross et al., 2013). During the first wave of COVID-19, nearly 80% of students in one recent study self-reported using social media to cope with COVID-related stress (Prowse et al., 2021). Evidence of a stress–screens association specific to social networking did not emerge in our data using device-logged records of time spent on apps such as TikTok and Instagram. Social networking may have instead brought people closer together by facilitating social connection during the pandemic, mitigating some negative effects of heightened stress. Indeed, emerging research shows that maintaining online social connection during the pandemic buffered against negative mental health and well-being effects, such as loneliness, anxiety, and stress (Beaunoyer et al., 2020; Stuart et al., 2021; Wiederhold, 2020).

### Prospective tests linking smartphone use to end-of-week mood states revealed only person-level rather than time-varying associations

We selected a 12-week longitudinal design for this study to allow finer-grained tests of associations between smartphone use and well-being than had previously been attempted in studies that collected device-logged reports of screen time. One study followed Prolific*-*recruited adults on a monthly basis in fall 2020 and linked iPhone Screen Time logs to depression and anxiety symptoms (Sewall et al., 2022). Across dozens of model specifications, no meaningful links were found, either concurrently or prospectively. If smartphone use induces more negative mood states, we ought to see evidence of it in time-varying prospective tests across weeks or days, if not across months. Instead, our findings ruled out most time-varying effects and revealed only person-level associations that were largely similar across categories of app. With accumulating failures to detect meaningful time-varying, within-person links between smartphone use and well-being, it appears increasingly unlikely that there is a simple dose–response relationship between greater minutes or hours of use and outcomes such as worse mood, higher stress, or symptoms of ill mental health (Johannes, Masur, et al., 2021).

Under a dose–response assumption, if smartphone use—and especially social media use—is causing harm to young people, we should see that weeks of heavier use precede worse moods. This was not the case in the present study, nor was it the case in an earlier experience-sampling study of 63 adolescents in The Netherlands that queried teens multiple times per day about their active (posting) and passive (scrolling) use of Instagram, Snapchat, and other apps (Beyens et al., 2020). During the majority of survey prompts, adolescents did not feel better or worse when they viewed posts or stories of other people, including on Instagram. At the time of drafting this article, Facebook (now Meta) was embroiled in a scandal over hiding internal research suggesting that for some teen girls, Instagram makes them feel bad about themselves (Wells et al., 2021). A key design feature of the leaked internal research was that girls were asked if they think using Instagram makes them feel worse (vs. better or neutral; Kamenetz, 2021). Crucially, their findings reflected the views of a tailored subgroup of girls who were already experiencing negative body image. As one journalist pointed out, self-reporting in this context is problematic because young people are “already primed by media coverage, and the disapproval of adults, to believe that social media is bad for them” (Kamenetz, 2021, para. 5). This concern has motivated study designs using device-logged reports of smartphone use, in which associations with mental health and well-being have been negligible (Beyens et al., 2020; Ellis et al., 2019; Sewall et al., 2020, 2021; Shaw et al., 2020).

Our findings from the present study that only person-level variation in smartphone use was associated with mood favor the interpretation that online problems reflect offline vulnerabilities (Odgers & Jensen, 2020). Stress, negative mood, poorer well-being, and ill mental health in young people are a function of ongoing person–environment interactions that include smartphones as one context of daily life. Students in the present study who generally tended to spend more time on favorite apps were the ones whose moods tended to be more negative—not just in a given week but in general. This association is likely capturing challenges also occurring in offline social and developmental contexts, including home, school, peers, and work. For example, students who experienced more COVID-related social isolation in the fall of 2020 might have relied more heavily on their phones. Even so, the sizes of person-level effects we detected in this study were small: A student who spent on average 1 more hour per day on their most frequently used app reported happiness levels across weeks that were about one tenth of a standard deviation lower than those of a typical student.

### Limitations

Several limitations of the current study offer useful insights to assist future research in further refining our understanding of links between smartphone use and well-being. First, this study was a pilot project that was limited to students recruited online via our local undergraduate participation pool and Prolific.co. These constraints left us with a predominantly female and predominantly White sample with a modest degree of retention week to week. Because of the gender imbalance, we elected not to pursue our preregistered analyses involving gender differences in time-varying stress–screen and screen–mood associations.

Second, participants in this sample were exclusively iPhone users, a large but incomplete cross-section of students with smartphones who may differ in their patterns of use from Android users. At least two studies have suggested moderate socioeconomic differences between iPhone and Android users (Götz et al., 2017; Gower & Moreno, 2018), which calls for the inclusion of both platforms in future screen time research. However, an EMA study of young adult Android users found no associations between momentary well-being and smartphone use (Johannes, Meier, et al., 2021), increasing our confidence in the generalizability of our findings.

We were also unable to rule out the possibility that at least some of the times logged in students’ Screen Time reports reflected their use of more than one device (iPhone, iPad, MacBook). Apple products allow for aggregating screen time estimates across devices via iCloud, and this function may have been enabled for some people. However, the frequently used apps depicted in Figure 2 are heavily weighted toward apps used exclusively or predominantly on smartphones, so multidevice Screen Time reports do not appear common in this sample.

Third, our models predicting end-of-week mood from smartphone use in the preceding week reflected students’ mood reports on Saturdays only and might not generalize to other days of the week. More generally, timing of smartphone use throughout the day (e.g., late night usage, usage that interrupts other activities) might be differently linked to stress and mood, but timing data were unavailable. Finally, data for this study were gathered during the first fall semester after universities had closed and shifted to online learning as a result of the COVID-19 pandemic, which may have impacted our findings. COVID-19 contributed to heightened stress levels among students as well as inflated time spent on screens compared with prepandemic times. Nevertheless, our results are in line with those of other studies linking device-logged screen time to well-being, both before and after the pandemic (David et al., 2018; Johannes, Sewall et al., 2020, 2022). Like those studies, however, ours is unable to provide insights into motives and reasons for using smartphones, nor how young people engage with their phones for good or ill. Qualitative methods analyzing rich conversations with young people about their smartphone use are needed to better understand how smartphones integrate into daily life.

### Conclusion

In sum, we established that weeks of higher stress were not linked to heavier smartphone use, nor did weeks of heavier use precede worse moods at the end of a given week, using device-logged screen time records in a sample of undergraduates during the fall semester of 2020. We found only between-persons associations linking more average hours of use to poorer average moods and linking more average phone pickups and better average moods. We detected no meaningful app-specific effects and notably no evidence of time-varying associations between social media use and higher stress or worse moods. Our findings contribute to a growing scholarly consensus that time spent on smartphones tells us little about young people’s well-being (e.g., Beyens et al., 2020; Coyne et al., 2020; Ellis et al., 2019; Ferguson et al., 2022; Johannes, Masur, et al., 2021; Orben, 2020; Sewall et al., 2020, 2021; Shaw et al., 2020). This message bears repeating to a general public aware that teens and emerging adults use screens at rates much higher than those of other age cohorts (Pew Research Center, 2021) and fearful for their mental health. Numerous studies focusing on quantities of smartphone use have failed to produce evidence of a robust link between hours of use and well-being, suggesting that an overall shift in thinking is called for in this line of research. A promising direction for future work is to focus on identifying individual differences that heighten susceptibility to negative effects of smartphone use as well as differences that position some young people to derive emotional, social, academic, and professional benefits from smartphone use. Smartphones and other digital technology function as a core developmental context for young people that cannot be extinguished by placing arbitrary limits on hours of daily use. Understanding how young people interact with the world during those hours is key to harnessing the potential of young people’s digital environments to support their better health and well-being.

## Transparency

*Action Editor:* Pim Cuijpers

*Editor:* Jennifer L. Tackett

*Author Contributions*

**Abigail H. M. Bradley:** Conceptualization; Data curation; Funding acquisition; Methodology; Project administration; Visualization; Writing – original draft; Writing – review & editing.

**Andrea L. Howard:** Conceptualization; Formal analysis; Funding acquisition; Methodology; Resources; Software; Supervision; Visualization; Writing – review & editing.

**Open Practices:** All data and materials have been made publicly available via OSF and can be accessed at https://osf.io/2gy63/. The design and analysis plans for the experiments were preregistered at OSF and can be accessed at https://osf.io/a9xpg. This article has received badges for Open Data, Open Materials, and Preregistration. More information about the Open Practices badges can be found at https://www.psychologicalscience.org/publications/badges.
